# Renal Outcome in Patients Undergoing Minimally Invasive Total Coronary Revascularization via Anterior Minithoracotomy Compared to Full Median Sternotomy Coronary Artery Bypass Grafting

**DOI:** 10.3390/jcm13185418

**Published:** 2024-09-12

**Authors:** Christian Sellin, Sarah Laube, Volodymyr Demianenko, Robert Balan, Hilmar Dörge, Peter Benoehr

**Affiliations:** 1 Department of Cardiothoracic Surgery, Klinikum Fulda gAG, University Medicine Marburg, Campus Fulda, 36043 Fulda, Germany; demcardio@gmail.com (V.D.); doerge@klinikum-fulda.de (H.D.); 2 Department of Anaesthesiology, Klinikum Fulda gAG, University Medicine Marburg, Campus Fulda, 36043 Fulda, Germany; sarah.laube2@klinikum-fulda.de; 3 Department of Cardiac Surgery, Klinikum Passau, 94036 Passau, Germany; robert.balan@klinikum-passau.de; 4 Department of Nephrology, Klinikum Fulda gAG, University Medicine Marburg, Campus Fulda, 36043 Fulda, Germany; peter.benoehr@klinikum-fulda.de

**Keywords:** minimally invasive cardiac surgery, coronary artery bypass grafting, CABG, acute renal failure, TCRAT

## Abstract

**Objective:** Renal dysfunction and acute renal failure after coronary artery bypass grafting (CABG) are among the main causes of increased mortality and morbidity. A sternum-sparing concept of minimally invasive total coronary revascularization via anterior minithoracotomy (TCRAT) was introduced with promising early and midterm outcomes in multivessel coronary artery disease. There are limited data regarding renal complications in patients undergoing the TCRAT technique. The present study analyzed renal outcomes in TCRAT compared to CABG via full median sternotomy (FS). **Methods:** We analyzed the records of 227 consecutive TCRAT patients (from September 2021 to June 2023) and 228 consecutive FS patients (from January 2017 to December 2018) who underwent nonemergent CABG. Following propensity score matching, preoperative baseline characteristics—including age, sex, diabetes mellitus, arterial hypertension, left ventricular ejection fraction, EuroSCORE II, preoperative serum creatinine, estimated glomerular filtration rate (eGFR), serum urea, and pre-existing chronic renal insufficiency—were comparable between the TCRAT (n = 170) and the FS group (n = 170). The examined postoperative renal parameters and complications were serum creatinine, eGFR, and serum urea on the first postoperative day. Moreover, serum creatinine, eGFR and serum urea at the time of discharge, postoperative ARF, and hemodialysis were investigated. Additionally, the duration of operation, CPB time, aortic cross-clamp time, ICU and hospital stay, ECMO support, rethoracotomy and in-hospital mortality were analyzed. The parameters were compared between groups using a Student’s *t*-test or Mann–Whitney U test. **Results:** The duration of operation (332 ± 66 vs. 257 ± 61 min; *p* < 0.05), CPB time (161 ± 40 vs. 116 ± 38 min; *p* < 0.05), and aortic cross-clamp time (100 ± 31 vs. 76 ± 26; *p* < 0.05) were longer in the TCRAT group. ICU (1.8 ± 2.2 vs. 2.9 ± 3.6 days; *p* < 0.05) and hospital (10.4 ± 7.6 vs. 12.4 ± 7.5 days; *p* < 0.05) stays were shorter in the TCRAT group. There were no differences between groups with regard to the renal parameters examined. **Conclusions:** Despite a prolonged duration of operation, CPB time, and aortic cross-clamp time when using the TCRAT technique, no increase in renal complications were found. In addition, ICU and hospital stays in the TCRAT group were shorter compared to CABG via full median sternotomy.

## 1. Introduction

Coronary artery bypass grafting (CABG) is the gold standard for the treatment of coronary artery disease (CAD) in multivessel disease and high anatomic complexity [1]. For the overwhelming majority of coronary artery surgery worldwide, full median sternotomy (FS) is used [2]. As a highly invasive therapeutic procedure, it is associated with complications such as restricted physical activity, wound-related issues, persistent thoracic pain, and poor quality of life. However, there has been rapid advancement in minimally invasive surgery, particularly in the areas of minimally invasive valve surgery and coronary revascularization [3,4,5]. Recently, a novel surgical approach for complete coronary revascularization in multivessel CAD via left anterior minithoracotomy (TCRAT) was introduced [6]. This technique has since been further developed into a less invasive standard method for arterial coronary bypass grafting that avoids sternotomy, utilizing established surgical techniques. The approach has demonstrated promising in-hospital and midterm outcomes [7,8,9]. This technique reduces surgical trauma and facilitates a faster postoperative recovery without compromising the fundamental principles of complete revascularization [7,8,9].

Despite advancements in surgical techniques and patient care, renal dysfunction and acute renal failure (ARF) continue to represent major concerns, contributing significantly to mortality, morbidity, extended intensive care unit (ICU), and hospital stays, as well as increased healthcare costs following cardiac surgery [10,11]. The incidence of ARF after open-heart surgery is in the range of 1–42%, while the incidence of renal failure requiring hemodialysis has been reported to vary in the range of 1–7% [10,12,13]. The resulting mortality varies in the range of 5–44% [14,15].

The association between CABG via FS and development of postoperative renal dysfunctions and complications has been established. Currently, there are no clinical data available on renal outcomes and complications following coronary revascularization using the TCRAT technique. According to a prolonged duration of the operation, of cardiopulmonary bypass (CPB), and of aortic cross-clamp time, a higher rate of renal complications could be anticipated.

The aim of the present study was to analyze the incidence of renal complications in comparison to CABG performed via FS.

## 2. Materials and Methods

### 2.1. Patient Selection and Data Collection

A total of 455 consecutive patients undergoing nonemergent isolated coronary bypass grafting were included in the study. Between September 2021 and June 2023, CABG via left anterior minithoracotomy with CPB using peripheral cannulation and cardioplegic cardiac arrest (transthoracic aortic cross-clamping) was performed in 227 patients (TCRAT group). In a historical cohort, from January 2017 to December 2018, CABG via FS with CPB using central cannulation was performed in 228 patients.

All patients were scheduled for surgery following a multidisciplinary heart team discussion [16], which included recommendations based on guideline indications [17] regarding which coronary arteries should be grafted. Anatomic complete revascularization was defined as the successful treatment of all significant coronary lesions with a visually estimated diameter stenosis of ≥50% in vessels with a reference vessel diameter of ≥1.5 mm [17]. Patients undergoing emergency procedures (defined as same-day catherization and operation), patients with significant atheromatous disease of the ascending aorta, patients with moderate or severe aortic regurgitation, patients undergoing reoperation, and patients who require dialysis preoperatively were not included. Data were derived from our internal quality assurance documentation and retrospectively extracted from patient records and presented as mean (± standard deviation), median, interquartile range (IQR), or as number (percentage).

Renal parameters and complications examined were estimated glomerular filtration rate (eGFR), serum creatinine, and serum urea on the first postoperative day (postoperative), as well as serum creatinine, eGFR, and serum urea at the time of discharge, postoperative ARF, and hemodialysis. In 2005, the Acute Kidney Injury Network (AKIN) proposed improvements to the RIFLE (Risk, Injury, Failure, Loss of kidney function and End-stage renal failure) criteria to make the detection of acute kidney injury clearer by adopting a lower cutoff point for variations in serum creatinine [13,15,18,19,20]. Based on this, postoperative ARF was defined as an increase in serum creatinine concentration of 0.3 mg/dL [15,21] or more. Moreover, we analyzed the duration of operation, CPB time, aortic cross-clamp time, ECMO support, rate of rethoracotomy, ICU and in-hospital stays as well as in-hospital mortality.

### 2.2. Statistical Analysis

To address selection bias in comparing the TCRAT and FS groups, propensity score matching (PSM) was performed. A propensity score was calculated for each patient using logistic regression analysis, incorporating ten preoperative variables: age, sex, arterial hypertension, left ventricular ejection fraction (LVEF), diabetes mellitus, EuroScore II, estimated glomerular filtration rate (eGFR), preoperative serum creatinine, serum urea, and preoperative chronic renal insufficiency. Finally, 170 pairs were successfully built in a 1:1 ratio (TCRAT n = 170, FS n = 170) through PSM [22], see Supplementary Materials.

Values for continuous variables are described as the mean ± standard deviation or percentage. The values between groups were compared using a Student’s *t*-test.

Non-normally distributed variables are additionally displayed as median and interquartile range and compared using a Mann–Whitney U test.

A *p* value of < 0.05 was considered statistically different.

All statistical analyses were performed using SPSS version 29.0.0.0 (IBM SPSS Statistics, Armonk, NY, USA).

### 2.3. Ethical Standards

This study was approved by the local ethics committee (University of Marburg, file number: 23-172 RS) and conducted in accordance with the ethical standards outlined in the 1964 Declaration of Helsinki and its subsequent amendments. Additionally, the study was registered in the German Clinical Trials Register (DRKS-ID: DRKS00032296).

### 2.4. Minimally Invasive Surgical Technique

The surgical technique of total coronary revascularization via left anterior minithoracotomy and the preoperative evaluation has been described in detail [7,8,9].

The operation was conducted with the patient in supine position. The radial artery (RA) was harvested using a minimally invasive endoscopic technique with a reusable retractor (Bisleri Model, Karl Storz, Tuttlingen, Germany) and a bipolar radiofrequency vessel sealing system (LigaSure, Medtronic, Minneapolis, MN, USA). Saphenous vein grafts (SVG) were harvested atraumatically under direct surgical vision. Grafts were preserved in a solution containing iron chelators (TiPROTEC, Dr. Franz Köhler Chemie GmbH, Bensheim, Germany).

The chest was accessed through an anterior muscle-sparing minithoracotomy, approximately 7–8 cm in length, in the fourth intercostal space. A retractor (Small Thoracotomy Retractor, Delacroix-Chevalier, Paris, France) was inserted. The left internal mammary artery (LIMA) was identified and harvested as a pedicle under direct surgical vision, using long conventional surgical instruments (35 cm DeBakey forceps and a 15 cm electrocautery blade) along with a specialized retractor (MIDAccess IMA Retractor, Delacroix-Chevalier, Paris, France).

The pericardium was opened and tacked to the skin.

Heparin was administered intravenously at a dose of 400 U/kg. Peripheral arterial cannulation was performed via the right axillary artery using a 16 or 18 Fr OptiSite Arterial Perfusion Cannula (Edwards Lifesciences, Irvine, CA, USA). Percutaneous venous cannulation was achieved through the common femoral vein, with a 23 Fr venous cannula (Bio-Medicus, Medtronic, Minneapolis, MN, USA) placed in the right atrium under ultrasound guidance. Vacuum-assisted venous return was routinely employed during CPB to enhance cardiac decompression. The ascending aorta was encircled with a tape to facilitate its exposure towards the minithoracotomy site, enabling the placement of a small cannula for the infusion of blood cardioplegia and venting. Patients were maintained at normothermia during CPB. Aortic cross-clamping was achieved using a transthoracic aortic clamp (ValveGate DeBakey, Geister, Plymouth, MA, USA), which was introduced through a separate small skin incision along the anterior axillary line at the level of the second intercostal space. Diastolic cardiac arrest was induced by infusing antegrade cold blood cardioplegia (Dr. Franz Köhler Chemie GmbH, Bensheim, Germany) and was sustained with intermittent cold re-infusion every 15–20 min.

After achieving cardiac arrest and decompression, the left pulmonary veins and inferior vena cava were encircled with tapes. By applying tension to these tapes and rotating the heart, access to all coronary territories was facilitated. Coronary anastomoses were performed using conventional coronary surgical instruments and the standard technique with continuous 8-0 polypropylene sutures. The LIMA was anastomosed as an in situ graft to the left anterior descending artery (LAD). Subsequently, the RA and/or SVG was anastomosed to the LIMA as a composite Y-graft or T-graft, or directly to the ascending aorta.

### 2.5. Standard Full Sternotomy Technique

The harvesting of the RA and SVG did not differ from the technique described above.

The operation was performed in the supine position.

FS as a surgical approach was the main difference to the TCRAT technique. LIMA harvest was performed under direct surgical vision as a pedicle. Following the opening of the pericardium, heparin was administered intravenously at a dose of 400 U/kg. Central arterial cannulation (24 Fr optimized flow aortic cannula; Sorin Group Italia S.R.L., Mirandola, Italy) was performed via the ascending aorta, and central venous cannulation was performed via a two-stage cannula (32/40 Fr venous return cannula, dual stage, wire-reinforced tubing, RDS-61040, LivaNova, Inc., Arvada, CO, USA) in the right atrium. An antegrade cardioplegia cannula (11 Fr DLP aortic root cannula, Medtronic, Minneapolis, MN, USA) was placed in the ascending aorta.

During CPB, the patients were kept normothermic.

After aortic cross clamping (Morris Atraumata aortic clamp, AESCULAP AG, Tuttlingen, Germany), diastolic cardiac arrest was induced with infusion of antegrade cold blood cardioplegia (Dr. Franz Köhler Chemie GmbH, Bensheim, Germany) and maintained with intermittent cold re-infusion every 15–20 min.

Coronary anastomoses were performed with the standard anastomotic technique of running 8-0 polypropylene sutures. The LIMA was anastomosed as an in situ graft to the LAD. The RA and/or SVG conduit was anastomosed to the LIMA as a composite T-graft or Y-graft or to the ascending aorta.

## 3. Results

Baseline characteristics before PSM are presented in Table 1. After propensity score matching, there were no significant differences in preoperative baseline characteristics between the groups (Table 2).

Patients who underwent TCRAT had a significant longer CPB time, aortic cross-clamp time, and operation time. ICU and hospital stays were shorter in the TCRAT group compared to the FS group (Table 3).

There were no differences between the two groups in terms of the renal parameters examined. Moreover, the rate of rethoracotomies and ECMO support were not different when comparing the two groups examined. Postoperative renal parameters and complications are presented in Table 4.

## 4. Discussion

This study aimed to evaluate and compare postoperative renal outcomes and complications in patients undergoing minimally invasive CABG using the TCRAT technique with those receiving CABG via FS.

The incidence of postoperative ARF following cardiac surgery according to the AKIN definition and the RIFLE criteria is reported to be between 5 and 42%. In our study, we found a postoperative ARF in 29% in both groups. The rate of hemodialysis required postoperatively was low in both groups, with 0.6% in the TCRAT group and 1.2% in the FS group compared to the literature findings [10,12,13,23].

Various factors are possible as the reasons for postoperative ARF, discussed in what follows.

First, renal hypoperfusion as a consequence of non-pulsatile low-flow circulation state during surgery by extracorporeal circulation appears to play an important role in the development of postoperative ARF. The role of CBP with regard to renal dysfunctions or ARF is still controversial. Among controlled randomized studies comparing on-pump and off-pump CABG, the CORONARY trial as well as the ROOBY trial showed no difference between groups for renal failure after CABG [24,25]. The HEPCON trial compared minimally invasive CPB with conventional CPB and off-pump surgery. The authors found a temporary superiority with regard to acute tubular necrosis in the minimally invasive group and the off-pump group. In the long-term follow-up, no differences between groups were found [26]. Our data show no significant differences in terms of the examined renal parameters and complications in comparison between the two groups examined despite significantly longer operation times, bypass times, and aortic cross-clamp times in the TCRAT group. Therefore, the data presented are consistent with the results of the three studies mentioned. Our findings are also in line with the results of the study by Vandewiele et al. [27]. The authors found no difference in renal function in the comparison between MICS-CABG, a beating heart procedure in which the anastomoses on the anterior and lateral walls of the heart are performed under direct visualization through an anterolateral mini-thoracotomy without CPB, and conventional sternotomy with concomitant mitral and/or tricuspid valve surgery despite longer CPB and aortic cross-clamp time.

Secondly, temperature fluctuations and volume shifts, e.g., caused by greater blood loss, can have an influence on the renal blood flow. Prolonged renal hypoperfusion or ischemia can lead to acute tubular necrosis. Moreover, intravascular hemolysis occurs after blood contact with artificial surfaces and is associated with a liberation of hemoglobin, which leads to a reduction in nitrogen oxidation, afferent arterial vasoconstriction, and an increase in heme oxygenase 1 expression [13,28,29]. In our study, as there were no differences in the use of routine CPB between the groups examined, this factor plays a rather minor role. Especially, in both groups, all patients were kept normothermic and the same volume of priming was used.

Third, inflammation and oxidative stress, essentially caused by the surgical trauma, artificial surfaces and blood transfusions, are further important factors in the development of ARF. These factors can cause a massive inflammatory cell accumulation and complement activation [30]. There was no difference in renal outcome in our study, despite the prolonged duration of operation time, of CPB time, and of aortic cross-clamp time and more blood transfusions in the TCRAT group. The rate of rethoracotomies and ECMO support, often associated with increased blood transfusions and increased contact with artificial surfaces, were not different when comparing the two groups examined.

Fourth, potentially nephrotoxic substances and medications can play an important role in the development of postoperative ARF. In the cardiac surgical setting, antibiotics (glycopeptides, aminoglycosides) and blood pressure medications (e.g., ACE inhibitors) are particularly worth mentioning. Furthermore, the use of contrast dye plays an important role in preoperative diagnostics [31]. The use of potentially nephrotoxic substances and medications plays no role in the comparison of the two groups examined, since the preoperative diagnostics and medications used intra- and postoperatively did not differ in both groups.

## 5. Study Limitations

There are several limitations of our study.

First, this is a single-center, retrospective study evaluating a new surgical technique. Additionally, patient selection was somewhat restricted, as individuals with severe calcifications of the ascending aorta were not treated using the minimally invasive approach. Furthermore, the study’s findings are constrained by a relatively short follow-up period, and the comparison of surgical procedures was limited to in-hospital outcomes.

Additionally, there are notable differences between the two groups concerning the types of grafts employed, the number of distal anastomoses performed, and the proportion of complete arterial revascularization achieved. This suggests that the TCRAT group employed a modified revascularization strategy, emphasizing multiarterial and complete revascularization, in line with established guidelines [17]. In conclusion, this is the first report of postoperative renal outcome in patients undergoing minimally invasive (TCRAT) compared to full median sternotomy coronary artery bypass grafting. Despite the prolonged duration of operation, CPB time, and aortic cross-clamp time when using the TCRAT technique, no increase in renal complications was found. In addition, ICU and hospital stays in TCRAT were shorter compared to CABG via full median sternotomy.

## Figures and Tables

**Table 1:** Preoperative baseline characteristics before propensity score matching.

Variables	TCRAT (n = 227)	FS (n = 228)	*p* Value
Age (years)	66 ± 10 (32–88)	69 ± 10 (46–85)	0.012
Male, n (%)	199 (88%)	192 (84%)	0.2
Diabetes mellitus, n (%)	70 (31%)	96 (42%)	0.014
Hypertension, n (%)	225 (99%)	221 (97%)	0.5
LVEF (%)	49 ± 10	52 ± 12	0.053
EuroScore II	2.9 ± 2.6	3.8 ± 4.6	0.3
Preoperative serum creatinine (mg/dL)	1.0 ± 0.3	1.1 ± 0.4	0.001
Preoperative eGFR (mL/min/1.73 m^2^)	77 ± 19	69 ± 21	<0.001
Preoperative serum urea (mg/dL)	36 ± 12	40 ± 18	0.01
Preoperative chronic renal dysfunction, n (%)	23 (10%)	41 (18%)	0.018

Data are presented as mean (± standard deviations) or absolute values (percentage %). LVEF, left ventricle ejection fraction; eGFR, estimated glomerular filtration rate.

**Table 2:** Preoperative baseline characteristics after propensity score matching.

Variables	TCRAT (n = 170)	FS (n = 170)	*p* Value
Age (years)	68 ± 10 (32–88)	68 ± 10 (46–85)	0.5
Male, n (%)	146 (86%)	148 (87%)	0.8
Diabetes mellitus, n (%)	55 (32%)	55 (32%)	>0.9
Hypertension, n (%)	167 (98%)	166 (98%)	>0.9
LVEF (%)	50 ± 9	51 ± 9	0.6
EuroScore II	3.0 ± 2.6	2.8 ± 3.0	0.3
Preoperative serum creatinine (mg/dL)	1.0 ± 0.3	1.1 ± 0.4	0.5
Preoperative eGFR (mL/min/1.73 m^2^)	76 ± 19	75 ± 20	0.3
Preoperative serum urea (mg/dL)	36 ± 12	36 ± 14	0.8
Preoperative chronic renal dysfunction, n (%)	19 (11%)	19 (11%)	>0.9

Data are presented as mean (± standard deviations) or absolute values (percentage %). LVEF, left ventricle ejection fraction; eGFR, estimated glomerular filtration rate.

**Table 3:** Intraoperative and postoperative characteristics.

Variables	TCRAT (n = 170)	FS (n = 170)	*p* Value
Aortic cross-clamp time, min Median IQR	100 ± 31 83 41	76 ± 26 87.5 28	<0.001
CPB time, min Median IQR	161 ± 40 197 46	116 ± 38 100 42	<0.001
Operative time, min Median IQR	332 ± 66 327 79.3	257 ± 61 251 66.8	<0.001
Number of distal anastomoses	3.1 ± 0.8 (2–5)	3.7 ± 0.9 (2–5)	<0.001
Conduits LIMA RIMA RA SVG	168 (99%) 0 (0%) 128 (75.3%) 106 (62.4%)	169 (99%) 71 (41.8%) 9 (5.3%) 146 (85.9%)	0.563 <0.001 <0.001 <0.001
Complete arterial revascularization, n (%)	63 (37.1%)	25 (14.7%)	<0.001
Multiple arterial revascularization, n (%)	65 (38.2%)	57 (33.5%)	0.363
Complete anatomical revascularization, n (%)	162 (95.3%)	162 (95.9%)	0.792
ICU length of stay, days Median IQR	1.8 ± 2.2 1 1	2.9 ± 3.6 2 1	<0.001
Hospital stay, days Median IQR	10.4 ± 7.6 8 4	12.4 ± 7.5 10 4	<0.001

Data are presented as mean (± standard deviations) or absolute values (percentage %). CPB, cardiopulmonary bypass; LIMA, left internal mammary artery; RIMA, right internal mammary artery; RA, radial artery; SVG, saphenous vein graft; ICU, intensive care unit; min, minutes; IQR, interquartile range.

**Table 4:** Postoperative renal parameters and complications.

Variables	TCRAT (n = 170)	FS (n = 170)	*p* Value
Postoperative serum creatinine (mg/dL)	1.1 ± 0.4	1.1 ± 0.4	>0.9
Postoperative eGFR (mL/min/1.73 m^2^)	73.9 ± 25	75.3 ± 27.4	0.1
Postoperative serum urea (mg/dL)	41.2 ± 13.5	41.5 ± 20.0	>0.9
Serum creatinine at the time of discharge (mg/dL)	1.1 ± 0.5	1.1 ± 0.4	0.3
eGFR at the time of discharge (mL/min/1.73 m^2^)	73.9 ± 24.7	69.6 ± 23.9	0.6
Serum urea at the time of discharge (mg/dL)	45.7 ± 19.8	43.1 ± 19.7	0.4
Postoperative ARF, n (%)	49 (28.8%)	49 (28.8%)	>0.9
Postoperative hemodialysis, n (%)	1 (0.6%)	2 (1.2%)	0.6
ECMO support, n (%)	1 (0.6%)	2 (1.2%)	0.6
Rethoracotomy, n (%)	11 (6.5%)	9 (5.3%)	0.6
In-hospital mortality, n (%)	1 (0.6%)	2 (1.2%)	0.6

Data are presented as mean (± standard deviations) or absolute values (percentage %). ARF, acute renal failure.

## Data Availability

The data presented in this study are available on request from the corresponding author due to (specify the reason for the restriction).

## Supplementary Materials

The following supporting information can be downloaded at: https://www.mdpi.com/article/10.3390/jcm13185418/s1.

## Glossary of Abbreviations

AKIN	Acute Kidney Injury Network
ARF	Acute renal failure
CAD	Coronary artery disease
CABG	Coronary artery bypass grafting
CPB	Cardiopulmonary bypass
eGFR	Estimated glomerular filtration rate
FS	Full median sternotomy
LAD	Left anterior descending artery
LIMA	Left internal mammary artery
LVEF	Left ventricular ejection fraction
PSM	Propensity score matching
RA	Radial artery
RIFLE	Risk, Injury, Failure, Loss of kidney function and End-stage renal failure
SVG	Saphenous vein graft
TCRAT	Total coronary revascularization via anterior thoracotomy
