# Supplementary material for: Renal Outcome in Patients Undergoing Minimally Invasive Total Coronary Revascularization via Anterior Minithoracotomy Compared to Full Median Sternotomy Coronary Artery Bypass Grafting
Source: J Clin Med. 2024 Sep 12;13(18):5418. doi: 10.3390/jcm13185418 (PMC11432697; doi:10.3390/jcm13185418)

Supplementary Materials

Figure S1: Histogram PSM parameter age

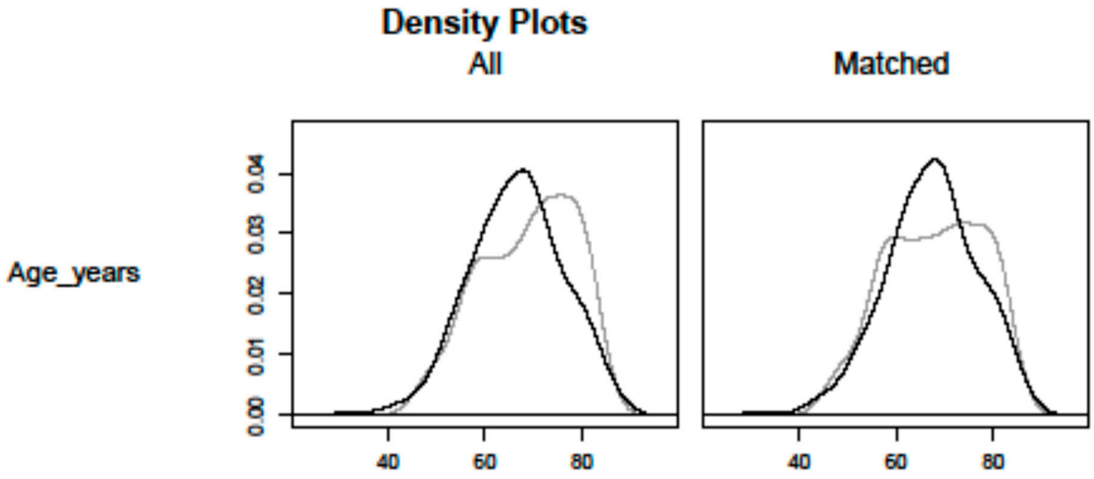

Figure S2: Histogram PSM parameter sex

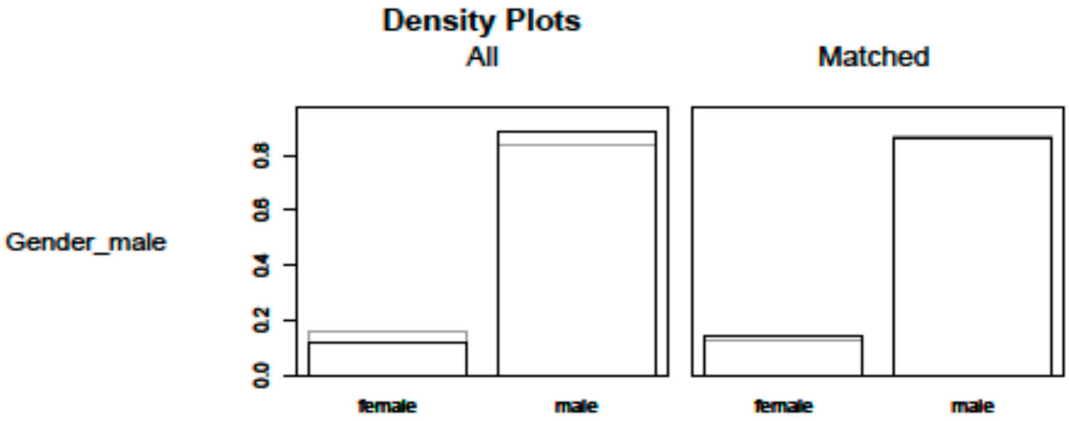

Figure S3: Histogram PSM parameter diabetes mellitus

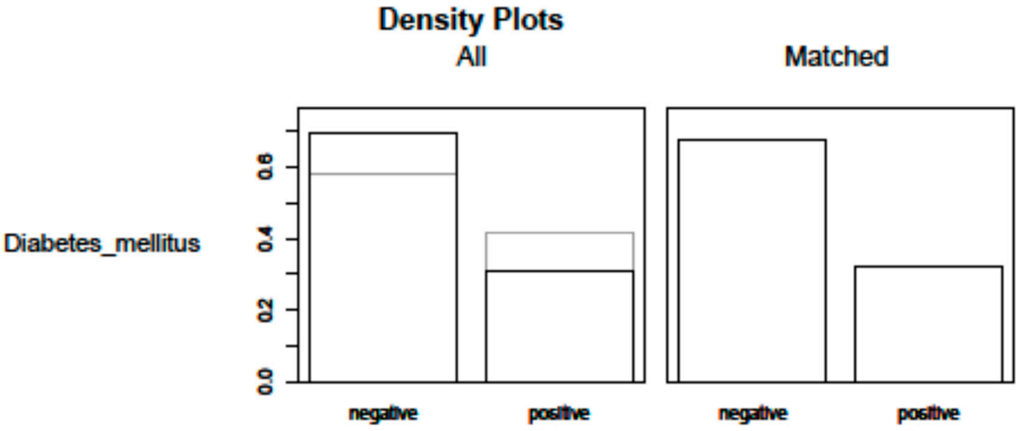

Figure S4: Histogram PSM parameter arterial hypertension

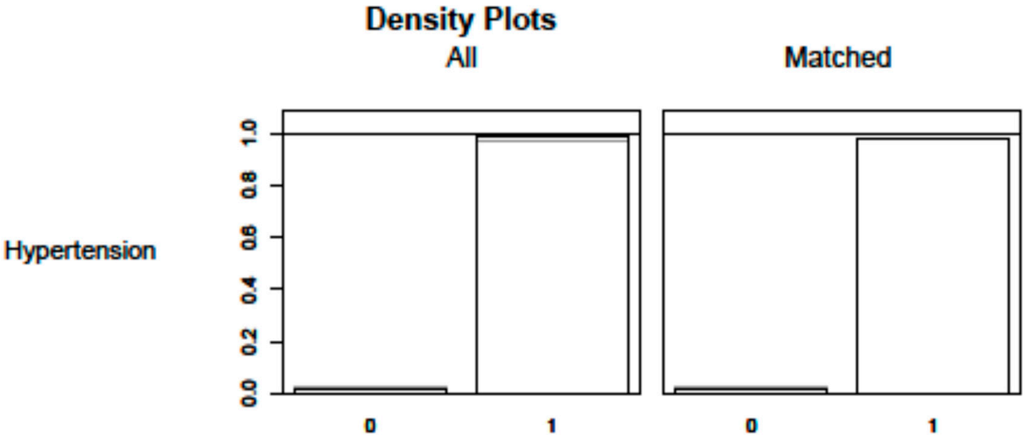

Figure S5: Histogram PSM parameter left ventricular ejection fraction

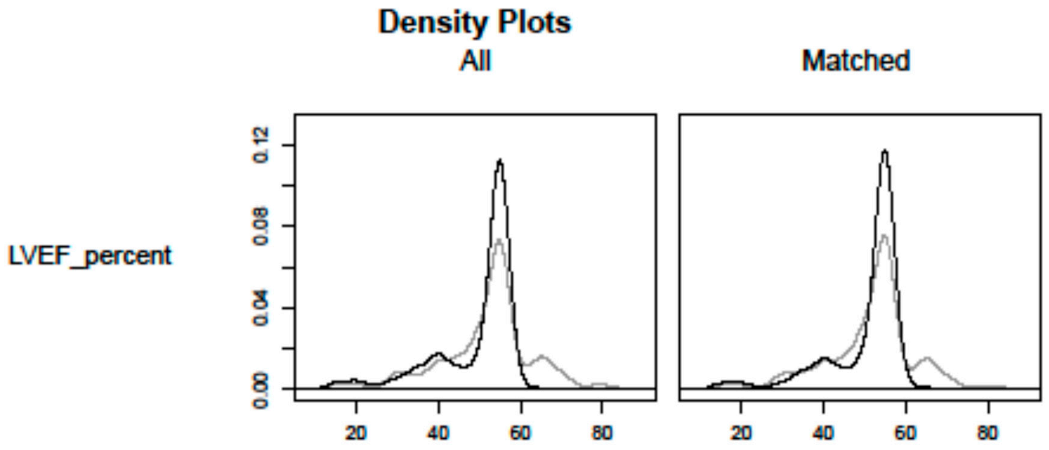

Figure S6: Histogram PSM parameter EuroScore II

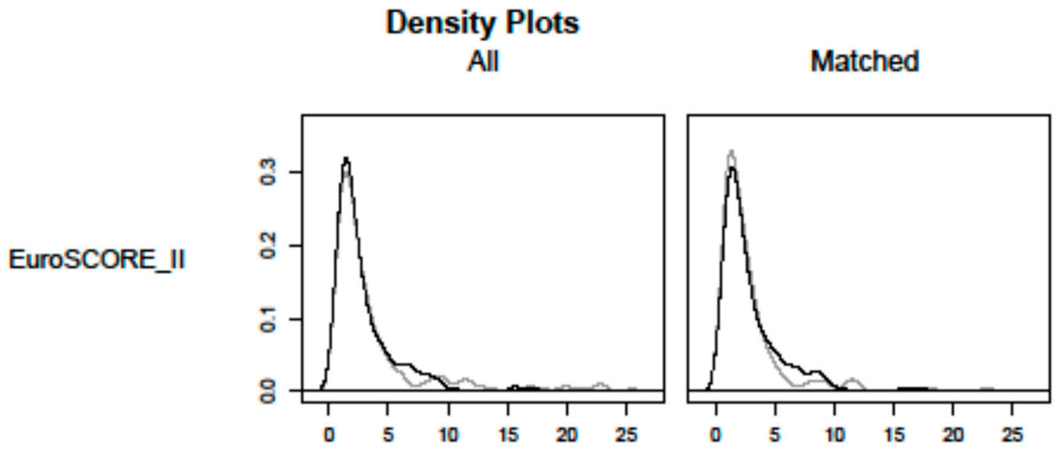

Figure S7: Histogram PSM parameter preoperative serum creatinine

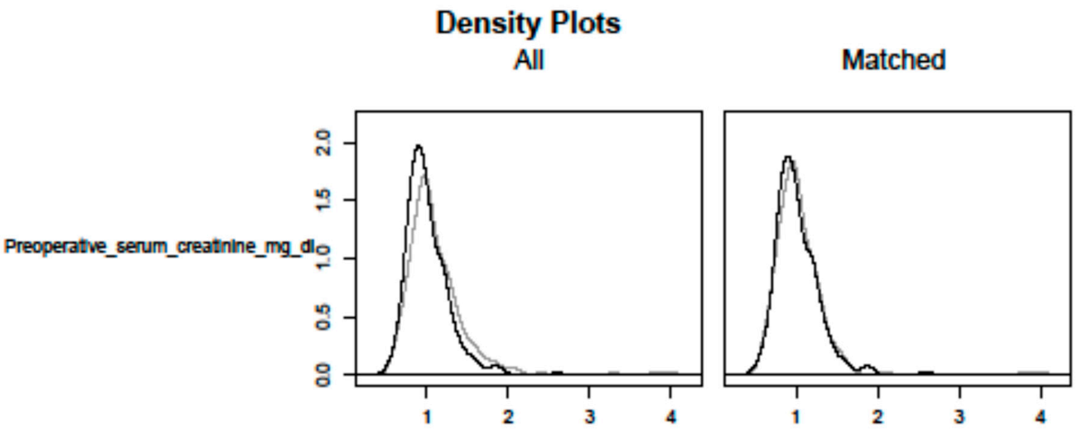

Figure S8: Histogram PSM parameter preoperative eGFR

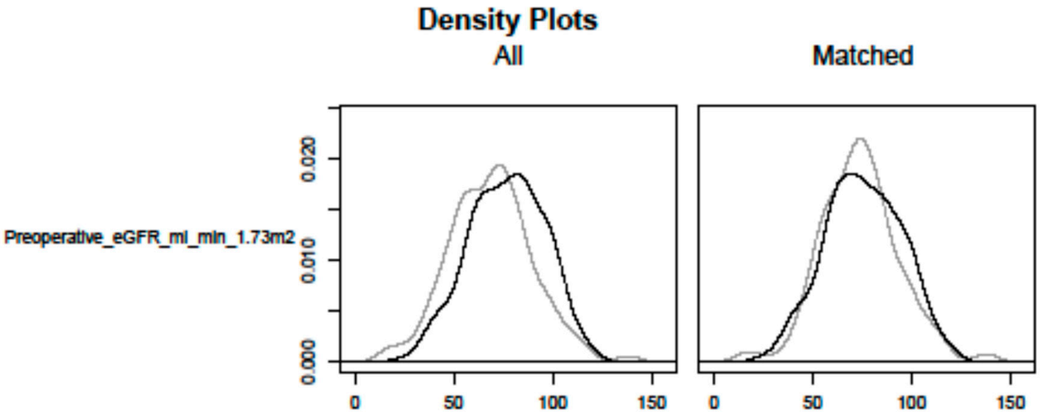

Figure S9: Histogram PSM parameter preoperative serum urea

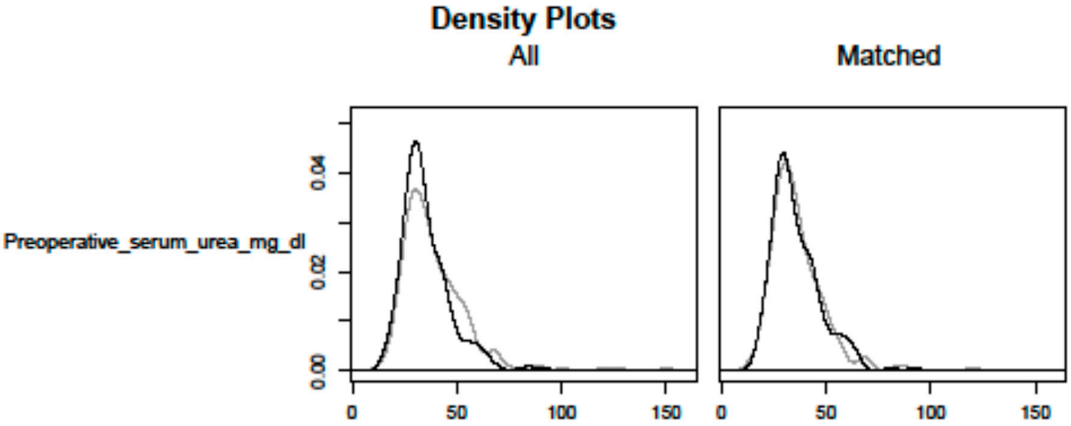

Figure S10: Histogram PSM parameter pre-existing chronic renal insufficiency

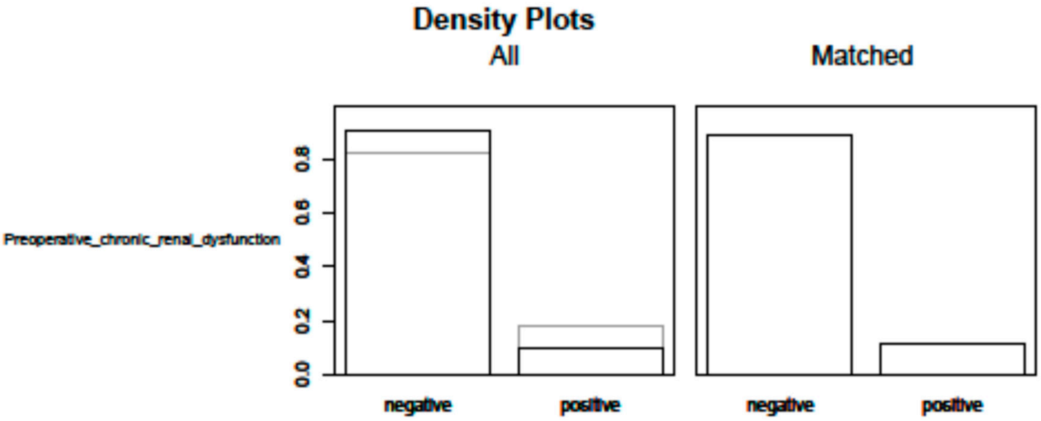

Supplement: Supplementary file 1 [file jcm-13-05418-s001.zip › jcm-3163125-supplementary.pdf]
